# Up all night on a *redeye* flight

**DOI:** 10.7554/eLife.02087

**Published:** 2014-02-04

**Authors:** Leslie C Griffith

**Affiliations:** 1 **Leslie C Griffith** is an *eLife* reviewing editor, and is in the Department of Biology, Volen Center for Complex Systems, National Center for Behavioral Genomics, Brandeis University, Waltham, United Statesgriffith@brandeis.edu

**Keywords:** Drosophila, sleep, acetylcholine signaling, cycling, sleepless/Lynx-1, homeostasis, *D. melanogaster*

## Abstract

A protein called RYE has a central role in the regulation of sleep.

**Related research article** Shi M, Yue Z, Kuryatov A, Lindstrom JM, Sehgal A. 2014. Identification of Redeye, a new sleep-regulating protein whose expression is modulated by sleep amount. *eLife* **3**:e01473. doi: 10.7554/eLife.01473

**Image** The sleep-regulating protein RYE is an output of the sleep homeostat, which ensures that an organism gets enough sleep

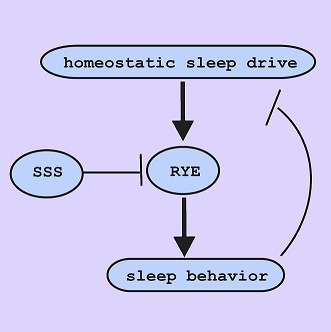

Experiments on invertebrate model organisms such as *Drosophila melanogaster* and *C. elegans* have provided a roadmap for exploring the molecular bases of a range of complex behaviours in humans. In the last decade, investigators have begun to point the power of genetics at one of the most important and mysterious behaviours of all: sleep. Behavioural studies in organisms from flies to humans imply the existence of a mechanism that ensures we get adequate amounts of sleep, but the molecular and cellular nature of this ‘sleep homeostat’ has remained elusive. Now, in *eLife*, Amita Sehgal and co-workers at the University of Pennsylvania—including Mi Shi as first author—have identified a gene that may help researchers get traction on this problem (Shi et al., 2014).

The neural mechanisms that control sleep are understood in only a very general way. In humans and other mammals, it is clear that there are a large number of brain regions that influence the normal daily sleep/wake transition and form the core of a ‘sleep switch’ that quickly moves the brain between wake and sleep states (Saper et al., 2010). This sleep switch is controlled by two mechanisms (Borbely, 1982). The first is the circadian clock, which schedules sleep to the part of the 24 hour day that is normal for that species. The second is the sleep homeostat, which measures how long the organism has been awake and adjusts the sleep switch to keep the organism asleep for an adequate length of time. The sleep homeostat therefore ensures that the total amount of sleep, when averaged over several days, is normal. Our understanding of the clock mechanism has been facilitated by the relatively simple nature of the circuits that control circadian rhythms in both vertebrates and invertebrates. The sleep homeostat is another matter: both its cellular and molecular nature are completely obscure.

Genetic screens in several fly labs have identified genes that alter the amount of time that animals spend asleep, but few if any genes that might be involved in the sleep homeostat have been discovered (Sehgal and Mignot, 2011). Shi et al. have now found that a certain point mutation in a gene called *redeye (rye)* reduces sleep time by over 50% due to a drastic decrease in the ability of the flies to remain asleep. This gene encodes a nicotinic acetylcholine receptor, and Shi et al. show that *rye* interacts with another gene, called *sleepless*, that was isolated by Sehgal and co-workers several years ago (Koh et al., 2008). Other experiments suggested that the *rye* mutation may be a dominant negative. Interestingly, expression of *rye* on a wild type genetic background did not increase sleep: this indicates that *rye* plays a necessary, but not sufficient, role in generation of sleep.

So far the *rye* story is pretty standard: *rye* is a broadly expressed gene that affects neuronal activity and has a sleep phenotype. Since the sleep state involves alterations in the activity of almost the whole brain, it is not surprising that generalized changes in neuronal activity are associated with changes in sleep. Consistent with this, genetics in invertebrate model organisms has largely (with a few notable exceptions) led to the identification of genes and signalling pathways that are very broadly active, and not the ‘sleep genes’ that one might hope to find.

However, the story got more interesting and sleep-specific when Shi et al. tracked the levels of *rye* messenger RNA and the RYE protein over time. RYE levels had a very pronounced and interesting daily pattern with two peaks—one at midday and one in the middle of the night. The levels of messenger RNA, on the other hand, were constant over the day. This means that RYE levels are being regulated after the gene has been transcribed to produce the messenger RNA. Significantly, RYE cycling was not necessary for the circadian clock to work properly because rhythmic behaviour was also observed in in *rye* mutants. Moreover, RYE cycling did not depend on the circadian clock because it was also observed in mutant flies that did not have a circadian clock. What did appear to affect RYE levels was the amount of prior sleep. A variety of short sleeping mutants—*sleepless* (Koh et al., 2008)*, fumin* (Kume et al., 2005), and *insomniac* (Stavropoulos and Young, 2011)—displayed elevated RYE levels that cycled during the day, which implied that the inability of these mutants to sleep stimulated the accumulation of RYE. But more importantly, six hours of sleep deprivation in wild-type flies caused an elevation of early morning RYE that coincided with increases in sleep generated by the homeostat. These data suggest that RYE is a marker of sleep drive (the increased desire to sleep after wakefulness), with the levels of RYE being controlled by the homeostat.

Does the identification of *rye* have implications for how sleep is generated? Probably, but this will rest on determining where RYE is required for sleep. If RYE regulates activity in many brain circuits, it may not be informative vis-à-vis the control of the sleep switch, but it might provide insight into how the switch is implemented. Will *rye* provide a tool to help us understand homeostasis? This seems the most exciting possibility. Having a molecular marker of the output of the homeostat provides a way into the homeostat. Working backward from how and where RYE levels cycle is a first step to revealing the mechanisms underlying this mysterious process.
